# Elevated Carbon Monoxide in the Exhaled Breath of Mice during a Systemic Bacterial Infection

**DOI:** 10.1371/journal.pone.0069802

**Published:** 2013-07-31

**Authors:** Alan G. Barbour, Charlotte M. Hirsch, Arash Ghalyanchi Langeroudi, Simone Meinardi, Eric R. G. Lewis, Azadeh Shojaee Estabragh, Donald R. Blake

**Affiliations:** 1 Departments of Medicine and Microbiology & Molecular Genetics, University of California Irvine, Irvine, California, United States of America; 2 Department of Chemistry and the Environmental Molecular Sciences Institute, University of California Irvine, Irvine, California, United States of America; Monash University, Australia

## Abstract

Blood is the specimen of choice for most laboratory tests for diagnosis and disease monitoring. Sampling exhaled breath is a noninvasive alternative to phlebotomy and has the potential for real-time monitoring at the bedside. Improved instrumentation has advanced breath analysis for several gaseous compounds from humans. However, application to small animal models of diseases and physiology has been limited. To extend breath analysis to mice, we crafted a means for collecting nose-only breath samples from groups and individual animals who were awake. Samples were subjected to gas chromatography and mass spectrometry procedures developed for highly sensitive analysis of trace volatile organic compounds (VOCs) in the atmosphere. We evaluated the system with experimental systemic infections of severe combined immunodeficiency *Mus musculus* with the bacterium *Borrelia hermsii*. Infected mice developed bacterial densities of ∼10^7^ per ml of blood by day 4 or 5 and in comparison to uninfected controls had hepatosplenomegaly and elevations of both inflammatory and anti-inflammatory cytokines. While 12 samples from individual infected mice on days 4 and 5 and 6 samples from uninfected mice did not significantly differ for 72 different VOCs, carbon monoxide (CO) was elevated in samples from infected mice, with a mean (95% confidence limits) effect size of 4.2 (2.8–5.6), when differences in CO_2_ in the breath were taken into account. Normalized CO values declined to the uninfected range after one day of treatment with the antibiotic ceftriaxone. Strongly correlated with CO in the breath were levels of heme oxygenase-1 protein in serum and HMOX1 transcripts in whole blood. These results (i) provide further evidence of the informativeness of CO concentration in the exhaled breath during systemic infection and inflammation, and (ii) encourage evaluation of this noninvasive analytic approach in other various other rodent models of infection and for utility in clinical management.

## Introduction

Body temperature, respiratory rate, and pulse rhythm were known to the ancients as informative parameters for diagnosing, staging, and monitoring disease. Subsequent advances in bedside monitoring were through technical enhancement of human capabilities and include the electrocardiogram, sphygmomanometer, and oximeter. A lengthening list of laboratory-based assays of substances and cells of the blood, urine, and other specimens provide additional information on the bodily status. However, assays of blood are seldom performed at the bedside, and it may be hours or days before results are known. When a patient’s status is rapidly changing, and supportive and therapeutic maneuvers are underway, such as in an intensive care unit, real-time data on the function of the cardiovascular, respiratory, and other systems is of critical importance for distinguishing salutary from counterproductive actions.

Ancient physicians also recognized the diagnostic utility of smelling the exhaled breath for tell-tale aromas, such as with hepatic or renal failure [1]. For some conditions unaided olfaction of the practitioner was sufficiently accurate for clinical decision making. These examples inspired efforts to extend human olfaction by means of instrumentation that provided better sensitivity and discrimination. While the potential value of breath analysis for disease-specific diagnosis, such as in diabetes [2], cystic fibrosis [3], or cancer [4], is undoubted, our interest instead is in compounds in the breath that might provide a unique dimension for the assessment and monitoring of patients with a variety of conditions.

To this end we initiated studies of experimental animals, but this required developing means for the noninvasive characterization of exhaled breath of individual mice. While there have been several examples of non-invasive breath analysis of humans (reviewed in [5]), nose-only methods have not to our knowledge been extended to laboratory mice who are awake and not intubated. A challenge has been the size of *Mus musculus*, which is ∼0.04% the mass of an adult human. In addition, for breath analysis to be feasible at the bedside, sampling durations should be in minutes if not seconds.

Here, we report non-invasive assessment of the exhaled breath of mice using analytical methods of atmospheric chemistry for trace gases. This approach was evaluated with a bacterial disease, *Borrelia hermsii* infection, with blood-borne dissemination and systemic inflammation [6]. After a survey for a large number of volatile organic compounds (VOC) in the exhaled breath of infected and non-infected mice, we identified the ratio of carbon monoxide (CO) to carbon dioxide (CO_2_) in the breath as an informative correlate of the state of infection of individual mice in this model and of the response to anti-infective therapy.

## Results

### Survey of Gases in Exhaled Breath of Infected Mice

**Figure 1:**
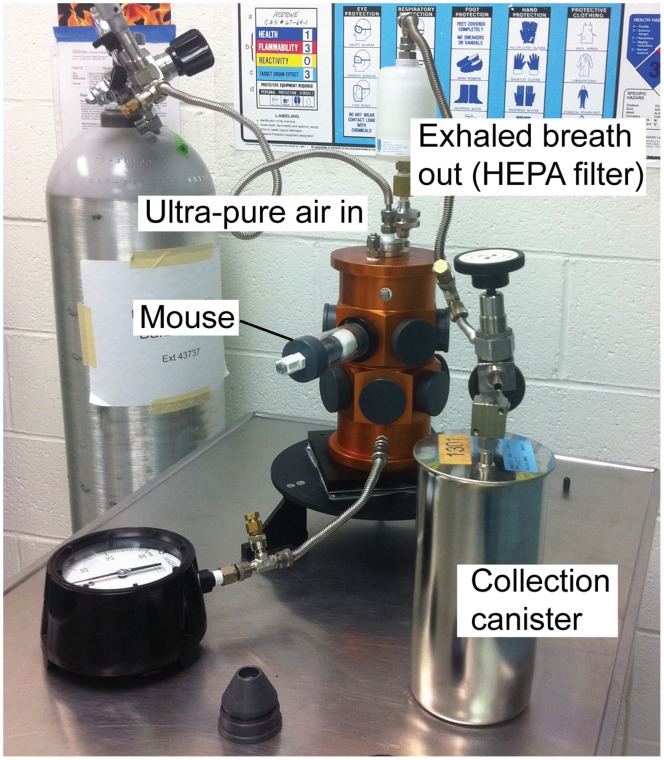
Assembled apparatus for collection of exhaled breath samples from groups or individual mice. Selected components of the apparatus are indicated.

In the first experiment 12 female and 12 male 5–7 week-old BALB/c-*scid* mice were first accommodated over 7 d of training to restraint without distress for up to 10 min at nose-only ports of a manifold for breath collection (Figure 1 and Figure S1 of Supplementary Information). Three mice at a time, one each at a port and grouped by sex, were positioned at the tower for breath sampling. Ultra-pure air at a flow rate of 1 L/min was flushed through the tower for 6 min, and then, while the air flow continued, a 2 min sample was collected. A blank sample of the ultra-pure air flushed through an empty port and chamber was collected at the same session. After breath samples of these groups of 3 were collected as a baseline on days −4, −2, and 0, the 24 mice were infected with *B. hermsii* on day 0 after the breath collection. Breath collections from the same groupings of 3 mice followed on days 3 and 5 with sacrifice on day 6. Counts of spirochetes in the blood reached their peak density of ∼10^7^ per ml by microscopy by day 5 in all infected mice. For days −4, −2, and 0, the 8 groups were counted as “uninfected”, and on days 3 and 5 they were designated as “infected”.

**Figure 2:**
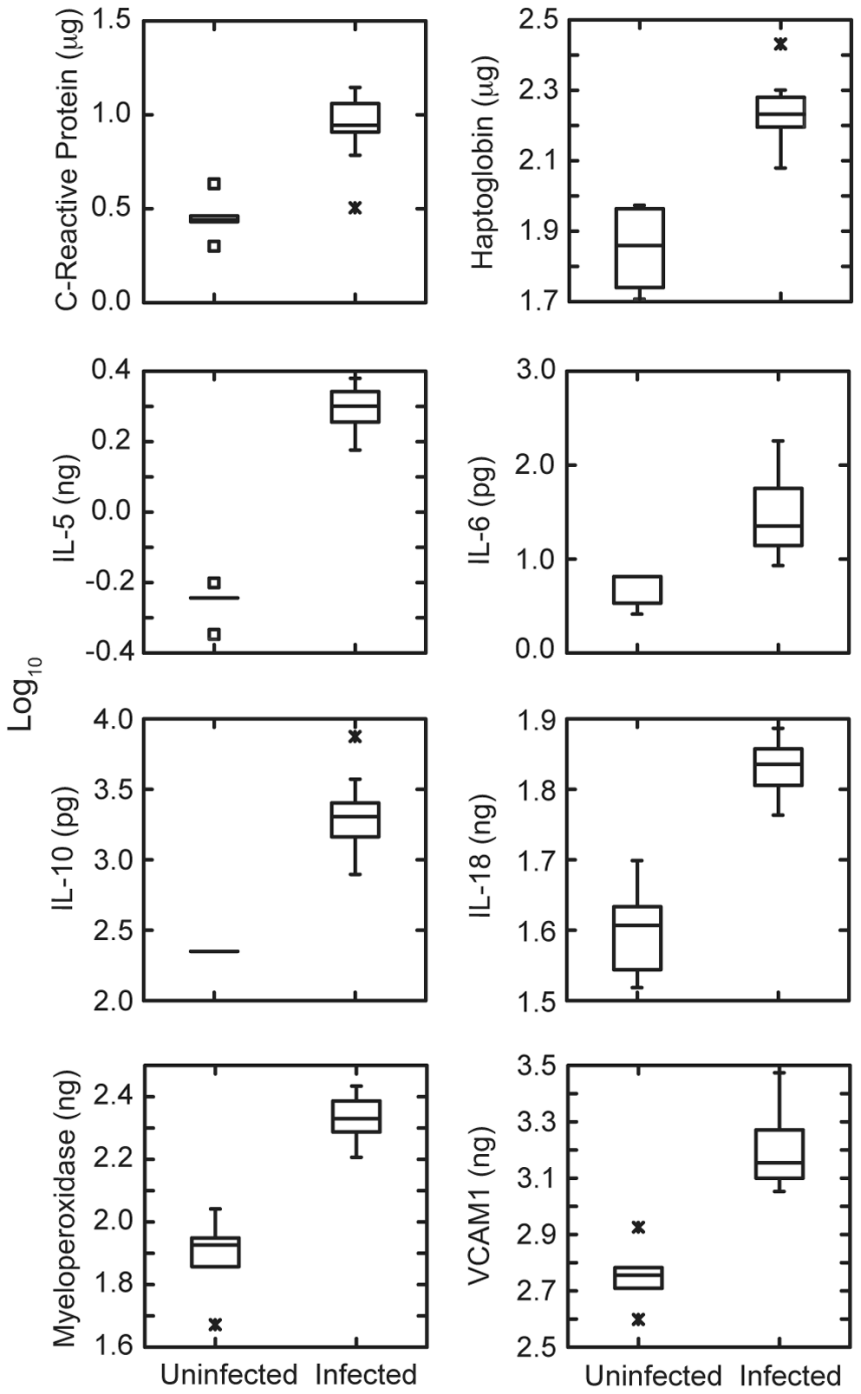
Selected acute phase reactants, cytokines, and other inflammation biomarkers in the plasma of BALB/c*-scid* mice that were uninfected (n = 6) or infected (n = 12) with *Borrelia hermsii*. Samples were obtained when bacteremia reached its peak in infected mice; analyte concentrations in wt per ml were determined by immunoassay as described in text. The graphs are box-whisker plots of log_10_-transformed values. Each box indicates the first and third quartiles, and the line inside the box is the median. The 1.5× interquartile range is indicated by the vertical line (whiskers) bisecting the box, and values outside this range are indicated by asterisks. If the result for a biomarker was “undetected”, we report the lower limit of quantitation for that substance on the run as the concentration for that specimen. Abbreviations: IL, Interleukin; VCAM1, Vascular Cell Adhesion Molecule-1.

We assessed the inflammatory state or other host parameters in response to infection among these mice by measuring a variety of cytokines, chemokines, and acute phase reactants at the time of peak bacteremia. Figure 2 shows the differences in log-transformed concentrations of selected inflammation and innate immunity markers in the sera of 12 infected mice on day 6 and in 6 uninfected, age- and sex-matched BALB/c-*scid* mice. There were raised concentrations of acute phase reactants, such as C-Reactive Protein and haptoglobin, and pro-inflammatory cyokines, such as Interleukin-6, as well as anti-inflammatory cytokines, such as Interleukin-10. The full list of analytes and results are given in Table S1 of SI. There was not a significant difference between 6 male and 6 female infected mice in these or other analytes after multiple testing correction.

For 16 blanks of air collected over the same sampling sessions, the mean concentrations of CO_2_ and CO were 0.036 (0.035-o.036)% and 89 (87–90) ppbv, respectively. For the 8 groups of uninfected mice on days −4 and −2, the mean total CO_2_ and CO concentrations in the 16 collected samples were 0.48 (0.41–0.55)% and 115 (108–121) ppbv. Thus, for uninfected mice ∼90% of the CO_2_ and ∼20% of the CO in the sample was attributable to the breath.

Seventy-three gases were detected and quantifiable in >90% of the samples (Table S2 of SI). For the large majority there was no discernible difference between the breath of infected and uninfected mice after correction for multiple testing. Carbon monoxide (CO) was the only compound that was significantly elevated in its total concentration in the 4 groups of infected mice on day 5 (196 [179–229] ppbv) in comparison to the combined 3 baseline samples from the 8 groups of uninfected mice (117 [113–126] ppbv) (*p*<0.0001).

### Relationship between CO and CO_2_ in the Breath

**Figure 3:**
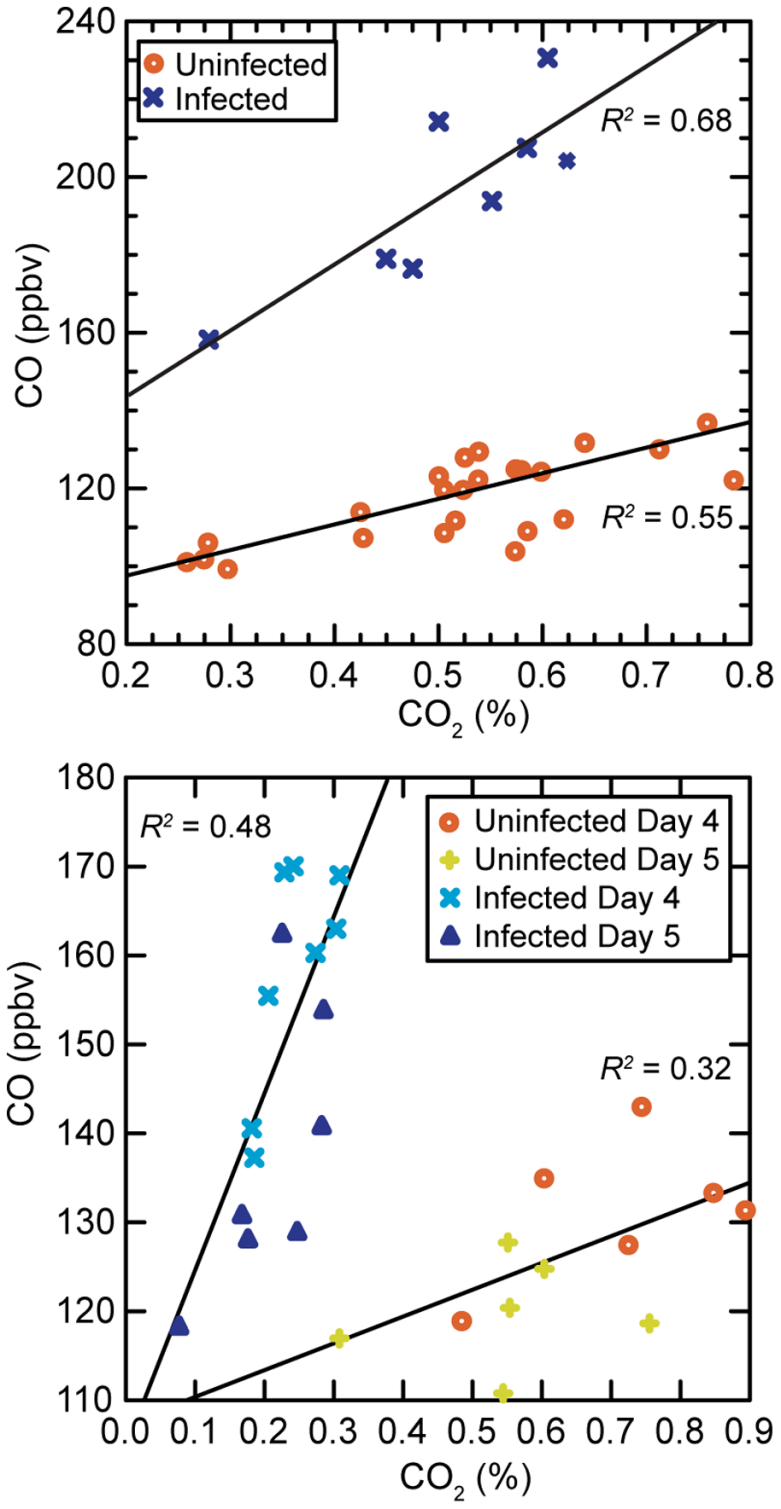
Least-squares linear regressions of carbon monoxide (CO) in parts per billion volume (ppbv) on total carbon dioxide (CO_2_) in % for samples of collected breath from mice infected with *B. hermsii* and uninfected controls in two experiments. Coefficients of determination (*R^2^*) for each group are shown in the figures. Upper panel: Eight groups of 3 BALB/c-*scid* male and female mice sampled on days −4, −2, and 0 (uninfected; total of 24 determinations) and injected with *B. hermsii* on day 0 after sampling. Mice were sampled again in the same groups on days 3 and 5. The plot shows the day 5 values for 8 groups of mice (infected). The inset indicates the symbols for status of infection. The regression coefficients (95% confidence interval) for the combined data for days 4 and 5 were 57 (39–75) for uninfected mice (*F*_1,22_ = 43.2; *p*<10^−4^) and 175 (97–253) for infected mice (*F*_1,6_ = 30.4; *p* = 0.002). Lower panel: Six groups of 3 C.B-17-*scid* male mice infected on day 0 and 5 groups of 3 uninfeceted mice sampled on days 4 and 5. The inset indicates the symbols for status of infection and day of sampling. The regression coefficients for the combined data for days 4 and 5 were 30 (10–51) for uninfected mice (*F*_1,10_ = 11.3; *p* = 0.007) and 195 (131–259) for infected mice (*F*_1,10_ = 43.2; *p*<10^−4^).

Differences in total CO concentrations in the canister samples between the 8 groups of mice on day 5 of infection and when they were uninfected on days −4, −2, and 0 (n = 24) were more apparent when these values were plotted against total CO_2_ concentrations (Figure 3, upper panel). CO concentration correlated with CO_2_ under both uninfected and infected conditions but along different linear regressions. At a given CO_2_ concentration, the CO in the sample from of a group of infected mice was about twice that from an uninfected group with a similar CO_2_. We confirmed this relationship in another experiment with 6–8 week-old male C.B-17-*scid* (n = 24) that were infected with *B. hermsii* on day 0. Breath samples were collected on days 4 and 5 from these mice in 8 groups of 3 and from 18 uninfected mice in 6 groups of 3 (n = 18). The lower panel of Figure 3 is a plot of total CO on CO_2_ in the cannister by infection state and day of sampling. While some of the uninfected mice groups had total CO concentrations in the collection cannisters that were higher than collected samples from some infected groups, the CO_2_ levels in samples from uninfected mice were up to 3-f0ld higher than corresponding infection samples. These results indicated to us that CO concentrations should be interpreted in the context of the measure of CO_2_ in the sample.

**Figure 4:**
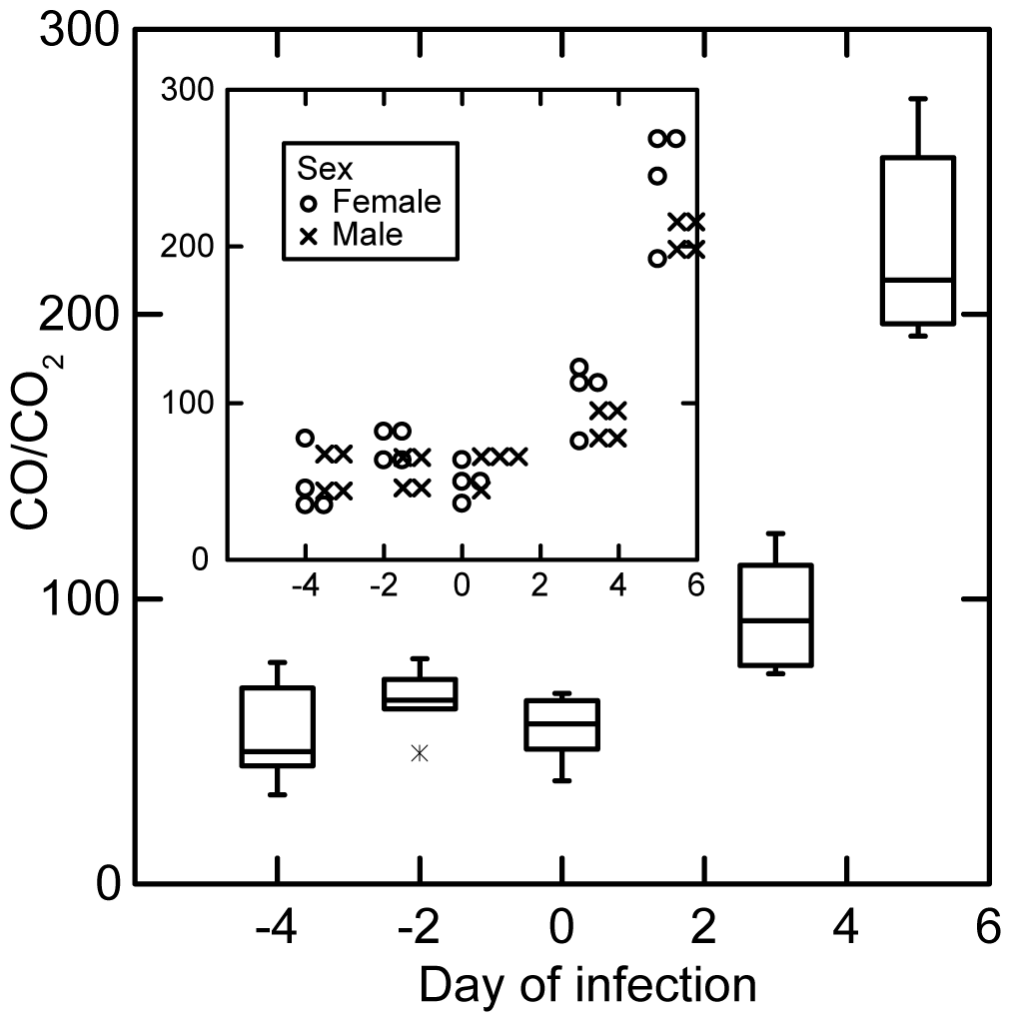
Box-whisker plots of normalized CO concentrations (CO/CO_2_) in the exhaled breath samples from 8 groups of 3 SCID mice infected with *B. * by day of infection. Breathhermsii samples were collected for 3 days before infection after the sampling on day 0. Inset, CO/CO_2_ by sex of group.

The collected sample comprised both the exhaled breath and the residual ultra-pure air ("background") that was used to flush the system during the collection. For subsequent analyses the net amount attributable to mice in the samples was calculated by subtracting the background concentrations from the total concentrations of CO and CO_2_. To take into account differences between mice in their ventilation during the sampling period, CO concentrations were normalized without units by dividing the net CO in ppbv by net CO_2_ as %. We represent this by the term “CO/CO_2_”. The values of CO/CO_2_ by day of the experiment for the 8 groups of BALB/c-*scid* mice before infection and then during infection by day is shown in Figure 4. The CO/CO_2_ values were stable on days −4, −2, and 0 before the injection and then markedly increased with progression of the infection over days 3 and 5. There was no apparent difference between groups of male and female mice in this response (inset).

To assess whether the SCID phenotype per se was associated with differences in CO and CO_2_ in the breath, we compared 16 samples from 5 groups of 3 uninfected female wildtype BALB/c mice and 12 samples from 5 groups of 3 uninfected, age-matched female BALB/c-*scid* mice over 3 collection sessions. The mean total CO and CO_2_ levels in the samples were 119 (114–124) and 0.53 (0.48–0.57)% for the immunocompetent mice and 115 (108–121) and 0.48 (0.41–0.55)% for the immunodeficient mice, respectively (*p*>0.2).

### Head Space of Cultures of *B. hermsii*

Given the high density of bacteria in the blood of infected mice, it was possible that the elevated CO concentrations were attributable wholly or in part to metabolic products by the bacteria themselves [7]. We investigated this by measuring differences in CO concentrations from the headspaces of sealed vessels with *B. hermsii* growing in its broth medium with 12% rabbit serum or with broth medium alone. The bacteria were grown to a density of 2×10^6^/ml at 37°C, and then divided into 3 aliquots of 5 ml each in 350 ml glass bioreactors. A control tube of medium without bacteria was incubated under the same conditions and then similarly dispensed in triplicate. The chambers were flushed with the ultra-pure clean air for 3 min at 1 L/min before being sealed and then incubated for another 24 h at 37°C, after which the cell densities were 10^7^/ml. The chamber was then connected through a stainless steel manifold to an evacuated canister for sample collection. The mean CO for the medium-only samples was 640 (628–652) ppbv, while the mean for the samples with cultured bacteria was 592 (555–630) (*p* = 0.07). This indicated that under conditions similar to the in vivo environment [8], the bacteria themselves were not producing CO, a finding consistent with the limited metabolic capacity of *Borrelia* species [9], [10].

### CO in the Exhaled Breath of Individual Mice

For the second set of experiments we initially used the same collection settings as for the first set. The daily blank values over 10 d of sampling averaged 0.036 (0.036–0.037)% for CO_2_, and 89 (86–91) ppbv for CO. For 66 samples from 20 uninfected mice over 10 d, the mean total concentrations in the collected samples were 0.126 (0.114–0.138)% for CO_2_, and 96 (95–97) for CO. The amount of CO_2_ attributable to respiration by a mouse, i.e. after subtraction of the background value, gave net CO_2_ values with unacceptably high variance for feasible sample sizes. As a modification, we reduced the flow of air from 1 L/min to 0.5 L/min and increased the collection period from 2 to 4 min. The blank CO and CO_2_ values over 12 d of collection were near-identical to the previous experiment, namely, 88 (86–90) ppbv and 0.036 (0.036–0.036)%. In contrast to the first experiment, for 100 samples from 18 uninfected mice over 12 d the mean total concentrations in the canisters were 106 (105–107) for CO and 0.389 (0.373–0.405)% for CO_2_, concentrations previously obtained only with groups of 3 or more mice.

By this modified protocol we sampled exhaled breath of eighteen 8–10 week-old, male C.B-17-*scid* mice, which weighed 19–23 gm, for the 3 d before infection was intiated on day 0 in 12 mice and then daily beginning on day 1. Six mice received injection of BSK II medium alone. Five infected mice were treated with the antibiotic ceftriaxone on day 4 after breath samples were collected and daily for 4 days thereafter. Five of 7 untreated infected mice were euthanized after day 4′s collection, and the other 2, as well as 2 uninfected mice, were euthanized after day 5. Four mice remained uninfected for the duration but received the same antibiotic treatment as for the infected animals. Table 1 summarizes data on 7 infected, 6 uninfected, and 5 treated mice at or before the time of sacrifice. At ∼15 genomes per *B. hermsii* cell [11], there was an average ∼10^7^ bacteria per ml of blood at time of sampling on the basis of quantitative PCR, as well as by microscopy. In comparison to uninfected animals, the infected mice lost weight and had enlarged livers and spleens.

**Table 1:** Characteristics of uninfected, infected, and treated SCID mice with systemic *Borrelia hermsii* infections.

Mouse status	Blood qPCR(genomes/ml)	Day −3 weight(gm)	Weight changeby day (mg)^a^	Liver(% body wt)	Spleen(% body wt)	Serum hemeoxygenase-1 (ng/ml)	Day 4breath CO/CO_2_	Day 8breath CO/CO_2_
Uninfected (NI) (n = 6)	<10^2^	22.48 (21.50 −23.47)	+55 (−3 – +113)	5.4 (5.1 – 5.7)	0.15 (0.13 – 0.17)	0.21 (0.05 – 0.16)	40 (32 – 49)	57 (43 – 71)
Infected (I) (n = 7)	7.7 (4.3 – 14) ×10^7^	23.40 (21.41 −25.39)	−223 (−161– −285)	6.2 (5.6 – 6.7)	0.86 (0.71 – 1.0)	1.79 (1.20 – 2.37)	279 (221 – 401)	–c
Treated (T) (n = 5)	<10^2^	22.92 (22.10 −23.74)	−100 (−42 – −178)	5.9 (5.2 – 6.6)	0.51 (0.47 – 0.56)	0.12 (0.10 – 0.15)	334 (267 – 401)	40 (34 – 47)
								
*p* I vs NI	–	0.45/0.83^b^	<0.0001/0.003	0.03/0.03	<0.0001/0.003	0.0004/0.003	<0.0001/0.003	–
*p* T vs I	–	0.59/0.94	0.04/0.04	0.59/0.69	0.004/0.007	0.0008/0.004	0.24/0.17	–
*p* T vs NI	–	0.71/0.85	0.005/0.02	0.14/0.10	<0.0001/0.006	0.02/0.02	<0.0001/0.006	0.09/0.14

a The number of days for each mouse were from day −3 until euthanasia (see text).

b *p* values by *t* test/Mann Whitney rank sum.

c -, not applicable.

**Figure 5:**
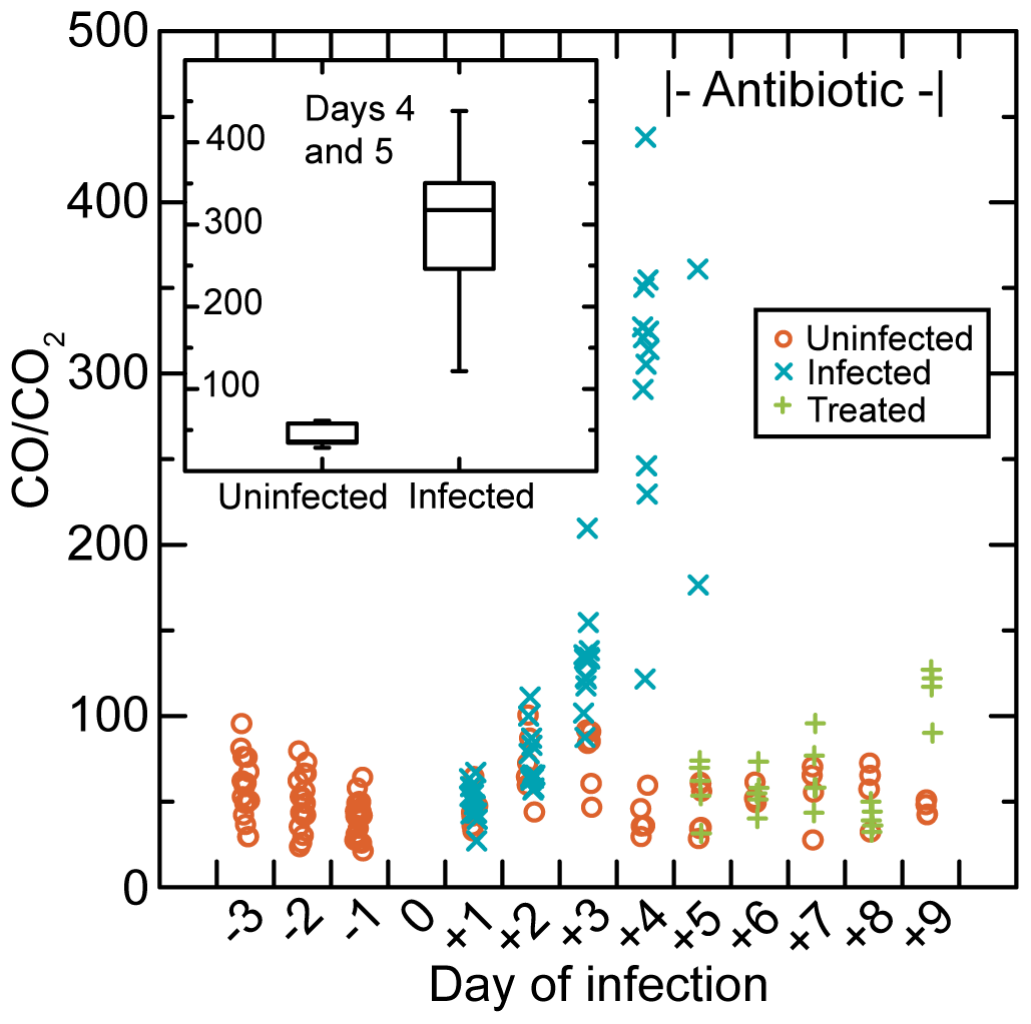
Daily normalized CO concentrations (CO/CO_2_) in the exhaled breath samples from 18 individual adult male SCID mice by state of infection (uninfected, infected, or treated) on day of study. *B. hermsii* infection was initiated on day 0 in 12 mice. On day 4 after the breath sampling, 5 infected mice were euthanized, 2 mice remained infected until euthanasia the next day, and 5 mice began treatment with the antibiotic ceftriaxone. Two uninfected mice were euthanized after day 5′s collection, and there is missing data for 1 treated mouse on day 9. The course for each mouse is given in Figure S2 of SI. Inset, box-whisker plots of CO/CO_2_ values for 6 uninfected mice, which were all sampled on days 4 and 5, and 12 untreated infected mice, 10 of which were sampled on day 4 only and 2 of which were sampled on day 5 as well.

Figure 5 shows the values for CO/CO_2_ for the mice by day and their status of uninfected, infected, or antibiotic-treated on the day of the study. The changes in CO/CO_2_ by day for each mouse are given in Figure S2 of SI. While there was little difference between individual mice before infections were initiated or among uninfected mice throughout the experiment’s course, CO/CO_2_ rose over days 1 to 4 to values 4-f0ld higher than for uninfected animals. On days 4 and 5, when bacteria were near or at their peak in the blood, the effect size for infected over uninfected mice was 4.2 (2.8–5.6) with a *r* of 0.91 (*p*<0.0001). Remarkably, CO/CO_2_ declined to the range of the uninfected mice within one day of the first dose of antibiotic. In contrast, infected mice who remained untreated continued to have elevated CO/CO_2_ on that day. By the end of treatment the pathogen burden in the blood had fallen from ∼10^7^ bacteria per ml to <10^2^ (Table 1).

### Heme Oxygenase

**Figure 6:**
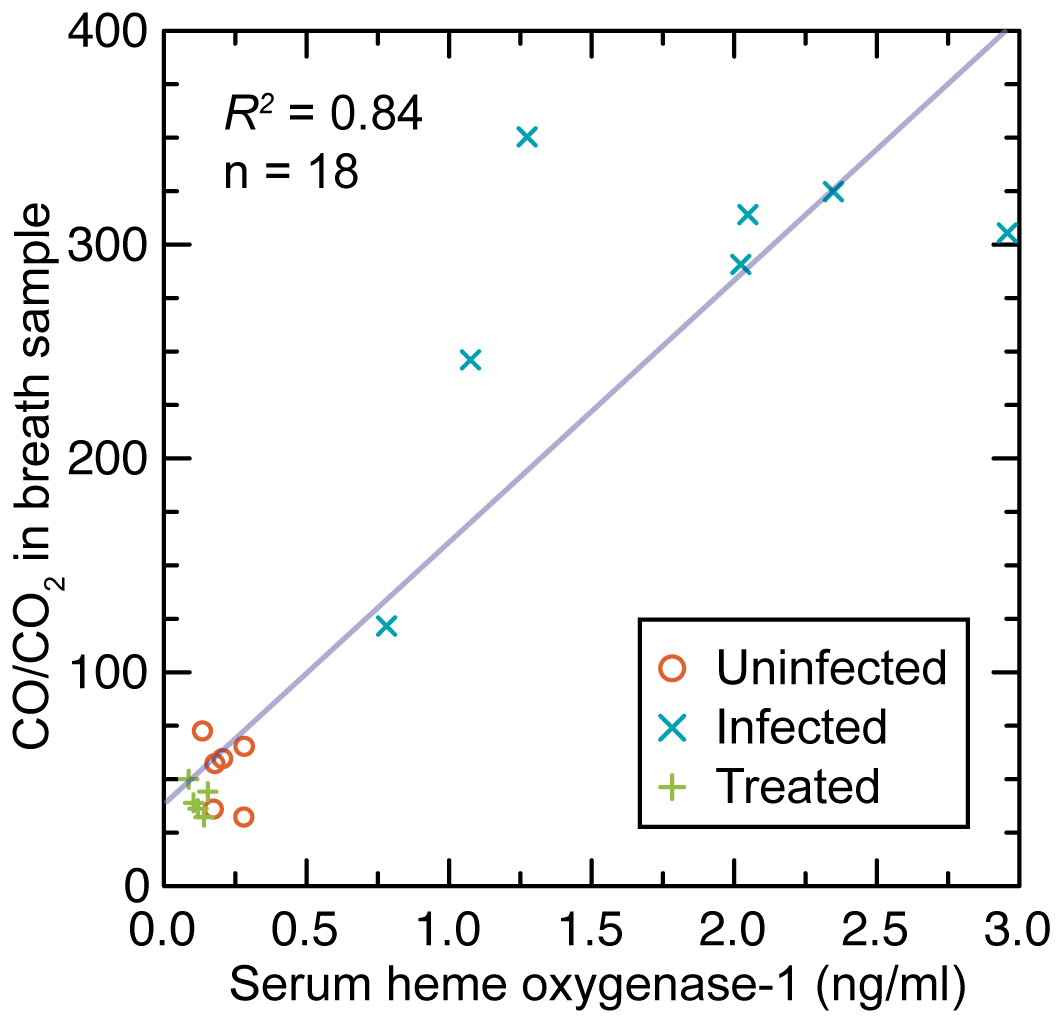
Scatter plot of normalized CO (CO/CO_2_) in breath samples on serum heme oxygenase-1 concentrations in 6 uninfected, 7 infected, and 5 treated male SCID mice at the time of euthanasia, as described in text and in Figure 5 legend. The least-squares linear regression line and the coefficient of determination (*R^2^*) are shown.

The source of endogenous CO is largely attributable to the action of the enzyme heme oxygenase-1 in tissues and the blood [7]. The contributions to CO production from lipid peroxidation or other processes are much less. We measured the concentration of heme oxygenase-1 in the serum, which was taken at the time of sacrifice from uninfected, infected, and treated BALB/c-*scid* mice, which were equally divided between males and females and which were studied under the same protocol as described above. Mean concentrations in ng/ml were 0.14 (0.01–0.28) for 4 uninfected mice, 0.65 (0.43–0.87) for 8 infected mice on day 4, and 0.05 (0.01–0.09) for 8 antibiotic-treated mice after 3 d. Among infected mice, females (0.58 [0.18–0.97]) and males (0.72 [0.48–0.96]) had similar values (*p*>0.5). We next compared heme oxygenase concentrations with CO/CO_2_ values for the male C.B-17-*scid* mice of Figure 5 and Table 1. Both CO/CO_2_ and heme oxygenase levels were elevated in untreated infected mice in comparison to uninfected or treated mice (Figure 6). When the serum heme oxygenase concentration was >0.5 ng/ml (n = 7), the CO/CO_2_ value was >100; when the enyzme concentration was ≤0.5 ng/ml (n = 12), the CO/CO_2_ value was ≤100 (*p*<10^−4^).

To investigate whether a source of the heme oxygenase enzyme in the serum were cells in the blood, we extracted mRNA from fresh whole blood of 6 infected female 5–7 week old C.B-17 *scid* mice on day 5 and 2 uninfected age- and sex-matched mice, produced cDNA, and then carried out quantitative PCR with primers that spanned introns in the HMOX1 gene and beta actin gene of mice. There were 84 (52–116) HMOX1 copies per 1000 beta actin copies in the extracted RNA from whole blood of infected mice, but only 1.6 (1.3–1.8) copies in uninfected mice (*p* = 0.04).

## Discussion

The study describes the development and application of a means for analyzing gaseous compounds present in the exhaled breath of individual mice that were non-invasively sampled for a few minutes under gentle restraint. Importantly, this was achieved without recourse to anesthesia and intubation of the mice [12], [13], which is both invasive and adds the presence of anesthetic gases, as well as VOCs from the procedure. It was also achieved with nose-only sampling instead of collections from whole animals in chambers, for hours at a time, and under conditions where CO_2_ levels can rise to 5% and feces and urine may be present [14], [15], [16].

The collected samples were effectively diluted by ∼10∶1 from flushing ultra-pure air, as estimated from the expected CO_2_ concentration of ∼5% in the exhaled breath and the observed values of ∼0.5% in samples from uninfected mice. So, there is scope for achieving greater sensitivity and shorter sampling periods with further improvements in design of the collection apparatus. Minimally diluted samples of the breath are more readily obtained from humans [2].

While total CO concentrations were about 2-fold higher in the samples from infected mice, the differences between infected and uninfected mice were up to 6-fold when differences between mice during the sampling were taken into account. Since the flushing rate with ultra-pure air was constant for each mouse sampled, irrespective of the ventilatory rate and metabolic state of the mouse, there would be relatively greater dilution of breath constituents for a mouse that was respiring less, as may have been the case for infected mice here (Figure 3), which typically have lower core temperatures during bacteremia [8] and may be less active. Another possible explanation is that the bacteremic mice had metabolic acidosis, which led to compensatory respiratory alkalosis with decreased CO_2_ concentrations in blood and exhaled breath. But in either case, normalizing net CO with net CO_2_ yields the CO/CO_2_ measure and corrects for individual differences in respiration. (For convenience we used the integer value of percent CO_2_ as the denominator in the calculation, because it yielded values that were similar in magnitude to net CO concentrations.) The normalization for CO_2_ concentrations presumably is also desirable for assessments of other gases in the breath, such as some VOCs, which tended to be present in lower concentrations in samples from infected animals (Table S2).

The study was limited to a single type of experimental infection, *B. hermsii*, and in an immunodeficient animal. We do not claim application to a general case. For that, various other models of disease in different hosts should be evaluated. Nevertheless, there are features of the model that may prove instructive for other infection and disease states. Use of SCID mice eliminated the contribution of adaptive immunity during the sampling period and provided for evaluation of the effect of an antibiotic alone. Except for some cytokines that largely derive from lymphocytes, the innate immune and other host responses of the mice, as the profiles of cytokines, chemokines, and acute phase reactants show (Figure 1 and Table S1), demonstrated the expected combination of both pro-inflammatory response, e.g. elevated IL-6, and anti-inflammatory response, e.g. IL-10, to a systemic infection by an extracellular bacterial pathogen. The elevations in the CRP and haptoglobin concentrations were also consistent with bacterial infection and an inflammatory state. Small rodents are natural hosts for *Borrelia* species and become ill during infection, as the weight loss and hepatosplenomegaly indicated, but do not suffer rapidly fatal infections [6]. In this respect, the model is more typical of endemic human infectious diseases than some experimental infections of mice with human-adapted pathogens.

This study began broadly in a discovery mode with application of an established analytic method of atmospheric chemistry with the capability to reveal in trace amounts hundreds of VOCs [17], [18]. Differences in VOCs in the exhaled breath may turn out to be informative for some infections, especially in the lung, such as in cystic fibrosis [3]. Moreover, the profile of compounds in the exhaled breath at this level of discrimination and quantification may provide an important correlate for changes in the respiratory tract or gut microbiomes. But initial findings here led us to focus on CO and then to the assessment that the source of the CO in the infected mouse was largely attributable to the animal and not the bacteria in this case. This was supported by findings of a positive correlation between heme oxygenase-1 concentrations in the serum and CO in the exhaled breath of uninfected, infected and untreated, and treated mice. Reverse-transcriptase qPCR identifed the blood itself, probably monocytes [19], as a source of heme oxygenase-1.

The induction of heme oxygenase expression and heightened production in CO in response or counter-response to sepsis, oxidative stress, and inflammation has garnered increased attention for several disease states (reviewed in [20], [21]), including malaria in animal models and humans [22], [23]. While understanding of the role of enodgenous CO as a modulator of inflammation remains incomplete, there is evidence that by affecting heme oxygenase-1 levels genetically or pharmacologically or by administering low-dose CO the outcome of a disease state can be altered (reviewed in [24], [25], [26], [27]). However, with the exception of small studies of heterogeneous patient populations [28], [29], there has been little study of CO in the human breath as a biomarker of disease. Our findings with single animals with masses of 20–30 grams provide a rationale for further studies of CO in the human breath as a biomarker that is informative for diagnosis, staging, and monitoring effects of resuscitation and therapy for a variety of conditions. Commercially-available, breadbox-sized devices for measuring CO and CO_2_ in the environment have sufficient sensitivity for the range of levels detected here and may be adaptable for real-time, non-invasive bedside use.

## Materials and Methods

### Sample Collection

A cylindrical inhalation chamber with 12 ports for nose-only collection of breath samples was obtained from In-Tox Products (Moriarty, New Mexico) (Figures 1 and S1). The restraints were Lexan tubes (In-Tox) with an inner diameter of 3.1 cm and a length of 9.0 cm. The tubes were connected to the tower via O-ring-sealed, positive flow-by nose pieces with outlets of 28 mm dia. The chamber was fitted with ¼” ultratorr connectors (Swagelok) at inlet and outlet ports. Connected to the inlet of the chamber by stainless steel flex tubing was a pressurized cylinder of ultra-pure air, which was collected at 10,000 feet elevation at the University of California’s Crooked Creek White Mountain Research Center. A Whatman HEPA filter was attached at the outlet. Additionally, a pressure gauge was connected at the base of the chamber for continuous pressure monitoring throughout the procedure. Samples of the exhaled mouse breath were collected using 2 L electropolished stainless steel canisters under controlled dynamic flow conditions, via connection to the HEPA-filter at the outlet of the chamber.

### Gas Analysis

CO was analyzed using a gas chromatograph (HP 5890) equipped with a flame ionization detector (FID) and a 3 m molecular sieve column (1/8″ O.D.). The first 3.5 min of effluent from the column were vented to the laboratory. At 3.5 min a 4-way switching valve directed the column outflow to a nickel catalyst (2% coating on Chromosorb G) where the CO reacted with H_2_ to form methane (CH_4_) that was detected by the FID set at 250°C. The oven temperature was kept at 60°C for 2 min, then raised to 110°C (at 70°C/min), and after 5 min returned to 60°C for a new analysis. The carrier gas was helium, the CO retention time was 5.3 minutes, and the amount of sample injected was 10 mL. The system for CO_2_ shared the manifold with the CO system. The sample was injected into a 6-foot long column (1/8″ O.D.; Alltech) packed with 80/100 mesh Carbosphere. CO_2_ was measured with the gas chromatograph equipped with a thermal conductivity detector set at 230°C. The oven temperature was kept at 150°C for 2.5 min, then raised to 220°C (at 70°C/min), and after 1.5 min returned to 150°C, for a new analysis. The carrier and reference gas used was helium; the CO_2_ retention time was 1.5 minutes. For CO, accuracy and precision were 1% and 2 parts per billion by volume (ppbv), respectively; corresponding values for CO_2_ were 1% and 3 parts per million by volume (ppmv). The analytical system for VOCs was a multi-column/detector combination gas-chromatographic system [17], [30], and consisting of 6 different column detector combinations from among 2 FIDs, 2 electron capture detectors, and a mass spectrometer. The different column/detector combinations allowed for the identification and quantification of several different classes of VOCs, including hydrocarbons, halocarbons, sulfur compounds, oxygenated gases, and alkyl nitrates, with detection limit of 10 parts per trillion by volume (pptv) for all the gases.

### Mouse Infections

*Mus musuclus* strains congenic with BALB/c with the severe combined immunodeficiency mutation (SCID) were CBySmn.C.B-17*-Prkdc^scid^*/J from Jackson Laboratory (BALB/c-*scid*) and C.B-17/Icr-*Prkdc^scid^*/IcrIcoCrl from Charles River Laboratories (C.B-17-*scid*). BALB/cJ mice were from Jackson Laboratory. Mice were housed in isolator cages under ABSL2 containment in an Association for Assessment and Accreditation of Laboratory Animal Care-approved facility, provided with autoclaved bedding and food (Harlan Teklad Global Soy Protein-Free Rodent Diet), were kept on a 12-h light-dark cycle, and received autoclaved distilled water ad libitum. Mice were examined and weighed daily. This study was carried out in strict accordance with the recommendations in the Guide for Care and Use of Laboratory Animals of the National Institutes of Health. The protocol was approved by the Institutional Animal Care and Utilization Committee of the University of California Irvine (protocol 2080-1999). Euthanasia and exsanguination was carried out under terminal anesthesia with isofluorane; all efforts were made to minimize suffering. *Borrelia hermsii* strain CC1 was passaged in mice or stored in plasma at −80°C as described [31]. Bacteria were cultivated in BSK II medium in tightly-capped polystyrene tubes [32]. Bacteria counts in culture medium or blood were determined by phase microscopy with a Petroff-Hauser chamber or by quantitative PCR (pPCR) with primers and probe for a region of the single-copy 16S rDNA gene as described [33]. DNA was extracted from 10 µl whole blood with DNeasy Blood Kit from Qiagen. With this volume of sample, the lower limit of quantitation of the qPCR assay was ∼100 genomes per milliliter of whole blood. Mice were inoculated intraperitoneally with ∼10^4^ bacteria in plasma diluted in medium to a volume of 100 µl or medium alone. Infection was monitored by microscopy of tail vein blood. Infected mice were either euthanized 1 day after bacteremia reached its peak density or treated with the antibiotic ceftriaxone (Sigma) in doses of 25 µg/g administered twice-daily subcutaneously [34]. During terminal anesthesia, blood was obtained with or without 1% sodium citrate, and the liver and spleen were removed and weighed. Plasma samples were subjected to bead-based immunoassays at Myriad RBM (Austin, TX) for the 59 analytes of the RodentMAP v. 2.0 panel (Table S1).

### Heme Oxygenase Protein and mRNA

Concentrations in ng/ml of mouse heme oxygenase −1 (HO-1) in serum diluted 1∶8 were measured in duplicate at OD 450 nm on a Synergy 2 microtiter plate reader (Biotek) with a sandwich enzyme-linked immunosorbent assay (Mouse Heme Oxygenase-1 EIA, Takara Bio). We developed a reverse-transcriptase qPCR for mouse heme oxygenase-1 gene (HMOX1) and beta actin transcripts. Total RNA was isolated from freshly obtained citrated blood using Qiamp RNA Blood Mini Kit (Qiagen), and cDNA synthesis was performed with a Maxima First Strand cDNA kit (Thermo Scientific). qPCR was performed in duplicate with qPCR Master Mix Plus for SYBRGreen (Eurogentec) in a Rotorgene 6000 (Corbett) thermal cycler. The forward and reverse primers that spanned introns of each were 5′GGTGATGGCTTCCTTGTACC and 5′AGTGAGGCCCATACCAGAAG for HMOX1 and 5′AGAGGGAAATCGTGCGTGAC and 5′CAATAGTGATGACCTGGCCGT for beta actin; products were 155 and 138 bp, respectively. The conditions were 10 min at 95°C and then 40 cycles of 15 s at 95°C and 1 min at 60°C. Standards were clones of the target in a plasmid vector. HMOX1 mRNA copies were normalized per 1000 copies of beta actin.

### Statistics

The 95% confidence intervals for means are given in parentheses or brackets. Parametric (*t* test) and non-parametric (Mann-Whitney rank sum) significance tests were carried out for continuous data and were 2-tailed. Either both or the highest *p* value of the two tests is given. Effect size was given by Cohen’s *d* statistic. The 2×2 contingency table test was exact and 2-tailed. Correction for multiple-testing was by the Bonferroni protocol. Linear regression by least-squares was carried out with Stata/MP v. 10.1 (Stata Corp., College Station, TX).

## Supporting Information

Figure S1 **Additional views of assembled apparatus for collection of breath samples.** In panel A, the breath sample is being evacuated into the collection canister. Panel B is a close-up of manifold tubes showing the adjustment for size of mouse. Panel C shows a mouse positioned at the nose-only port of the manifold and while ultra-pure air is flushing the unit.
(PDF)

Figure S2 **Normalized CO (CO/CO_2_) for 18 male** ***scid*** **mice by day of infection and by infection and antibiotic treatment condition of the experiment.** Infected mice were inoculated with *B. hermsii* on day 0 and euthanized on either day 4 or day 5 after that day’s breath collection. Treated mice were infected on day 0 and received ceftriaxone on day 4 after sampling. Uninfected mice likewise received ceftriaxone beginning day 4.
(PDF)

Table S1 **Analytes in the plasma of infected and uninfected mice.**
(PDF)

Table S2 **Carbon monoxide and volatile organic compounds in exhaled breath of mice, in parts per trillion by volume (pptv), except as noted.**
(PDF)
