# Supplementary figures and images for: Elevated Carbon Monoxide in the Exhaled Breath of Mice during a Systemic Bacterial Infection
Source: PLoS One. 2013 Jul 31;8(7):e69802. doi: 10.1371/journal.pone.0069802 (PMC3729689; doi:10.1371/journal.pone.0069802)

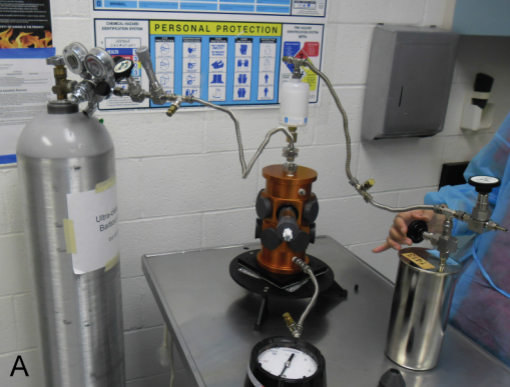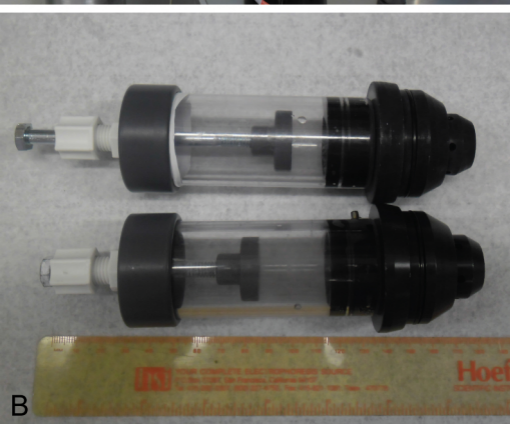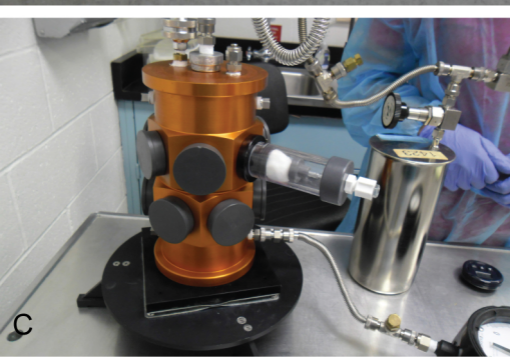

Supplement: Figure S1 — Additional views of assembled apparatus for collection of breath samples. In panel A, the breath sample is being evacuated into the collection canister. Panel B is a close-up of manifold tubes showing the adjustment for size of mouse. Panel C shows a mouse positioned at the nose-only port of the manifold and while ultra-pure air is flushing the unit. (PDF) [file pone.0069802.s001.pdf]

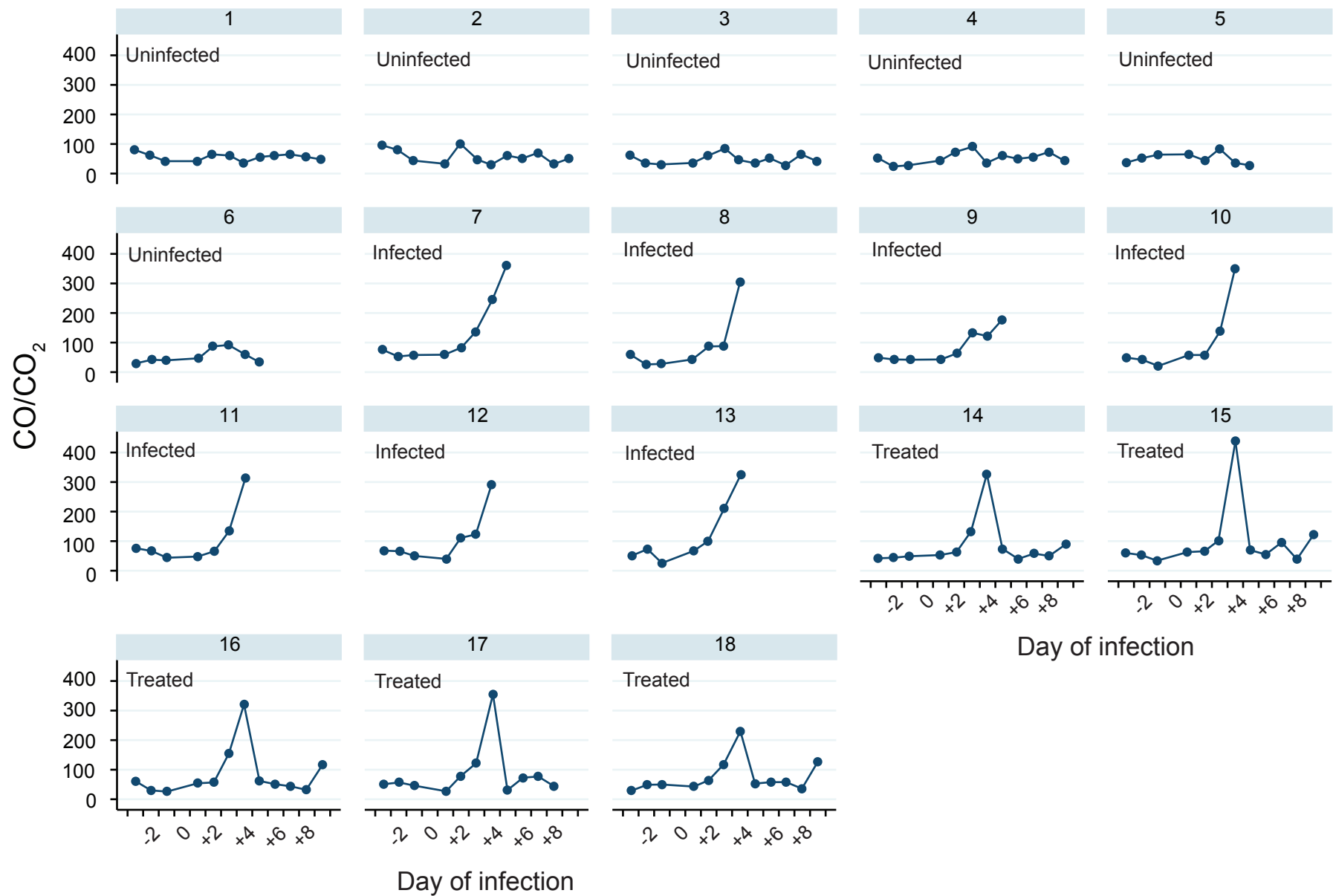

Supplement: Figure S2 — Normalized CO (CO/CO2) for 18 male scid mice by day of infection and by infection and antibiotic treatment condition of the experiment. Infected mice were inoculated with B. hermsii on day 0 and euthanized on either day 4 or day 5 after that day’s breath collection. Treated mice were infected on day 0 and received ceftriaxone on day 4 after sampling. Uninfected mice likewise received ceftriaxone beginning day 4. (PDF) [file pone.0069802.s002.pdf]
