# Supplementary material for: Elevated Carbon Monoxide in the Exhaled Breath of Mice during a Systemic Bacterial Infection
Source: PLoS One. 2013 Jul 31;8(7):e69802. doi: 10.1371/journal.pone.0069802 (PMC3729689; doi:10.1371/journal.pone.0069802)
Supplement: Table S1 — Analytes in the plasma of infected and uninfected mice. (PDF) [file pone.0069802.s003.pdf]

Table S1. Analytes in the plasma of infected and uninfected mice

Analyte[a]	Units/ml	Lower detection value	Infected (n = 12)			Uninfected (n = 6)			*t* test *p* value[b]
			Mean	95% confidence limits		Mean	95% confidence limits		
Apolipoprotein A-I	ug	2.4	115	81	149	209	171	248	4.0E-03
C-Reactive Protein	ug	0.9	9.2	7.6	10.9	2.9	2.3	3.5	1.2E-04
CD40	pg	4.5	212	-81	505	33	29	38	4.2E-01
CD40 Ligand	pg	414	1825	1189	2460	382	199	564	7.3E-03
Endothelin-1	pg	36	43	40	46	54	48	59	4.4E-03
Eotaxin	pg	5	602	440	763	734	574	893	3.3E-01
Epidermal Growth Factor	pg	9.3	41	36	45	47	41	53	1.3E-01
Factor VII	ng	28	67	64	70	58	52	63	2.7E-03
Fibroblast Growth Factor basic	ng	23	38	35	41	44	36	52	1.1E-01
Granulocyte Chemotactic Protein-2	ng	0.38	7.4	6.3	8.4	11.1	8.7	13.4	4.0E-03
Growth-Regulated Alpha Protein	ng	0.024	1.47	0.233	2.71	0.049	0.020	0.077	1.4E-01
Haptoglobin	ug	6	176	156	197	73	58	88	8.1E-06
Immunoglobulin	ug	5.2	13.7	9.8	17.6	24.0	20.7	27.3	4.1E-03
Interferon gamma	pg	23	20.7	15.8	25.5	12.0	5.9	18.1	5.2E-02
Interferon gamma Induced Protein 10	pg	14	375	277	472	209	73	345	6.9E-02
Interleukin-1 beta	ng	6	6.2	5.5	6.8	4.0	2.3	5.7	1.5E-02
Interleukin-5	ng	0.41	2.0	1.9	2.1	0.6	0.5	0.6	2.1E-10
Interleukin-6	pg	6.5	46.6	16.4	76.9	5.3	3.8	6.8	8.1E-02
Interleukin-10	pg	224	2437	1433	3440	224	224	224	8.3E-03
Interleukin-18	ng	4.3	68	65	71	40	35	45	8.4E-08
Leukemia Inhibitory Factor	pg	707	1066	955	1178	1204	997	1412	2.2E-01
Lymphotactin	pg	26	98	79	117	82	35	129	4.7E-01
Macrophage Colony-Stimulating Factor-1	ng	0.009	5.4	4.9	5.9	5.3	4.6	6.1	9.3E-01
Macrophage-Derived Chemokine	pg	44	552	426	678	580	472	688	7.8E-01
Macrophage Inflammatory Protein-1 alpha	ng	2.9	12.2	11.5	13.0	9.5	8.8	10.3	5.2E-04
Macrophage Inflammatory Protein-1 beta	pg	50	1980	1334	2627	96	56	136	1.1E-03
Macrophage Inflammatory Protein-1 gamma	ng	0.92	63	48	77	15	14	17	3.6E-04
Macrophage Inflammatory Protein-2	pg	5.9	243	30	457	10	5	15	1.5E-01
Macrophage Inflammatory Protein-3 beta	ng	0.22	4.2	3.7	4.8	3.2	2.7	3.7	2.7E-02
Matrix Metalloproteinase-9	ng	7.8	210	142	278	57	45	68	7.4E-03
Monocyte Chemotactic Protein 1	pg	5	705	496	914	41	8	74	5.2E-04
Monocyte Chemotactic Protein 3	pg	8.1	1316	919	1714	70	46	94	5.7E-04
Monocyte Chemotactic Protein-5	pg	3.9	136	81	192	17	13	21	1.0E-02
Myeloperoxidase	ng	0.6	217	197	237	81	64	98	2.2E-07
Myoglobin	ng	6.8	2100	1312	2889	2484	740	4227	6.6E-01
Serum Amyloid P-Component	ug	3.7	52	46	59	22	18	26	1.8E-05
Serum Glutamic Oxaloacetic Transaminase	ug	53	112	98	127	126	67	185	5.7E-01
Stem Cell Factor	pg	130	464	316	613	297	191	404	1.6E-01
T-Cell-Specific Protein RANTES	pg	0.076	0.045	0.031	0.059	0.043	0.013	0.072	8.9E-01
Thrombopoietin	ng	22	109	101	116	57	45	68	8.6E-07
Tissue Factor	ng	3.6	12.3	10.9	13.7	14.8	12.6	17.0	6.5E-02
Tissue Inhibitor of Metalloproteinases 1	ng	0.057	5.7	3.5	7.8	0.8	0.6	0.9	6.5E-03
Vascular Cell Adhesion Molecule-1	ng	2.8	1633	1316	1951	583	463	703	4.0E-04
Vascular Endothelial Growth Factor	pg	45	1077	611	1542	488	389	587	1.1E-01
von Willebrand factor	ng	15	101	86	117	45	35	54	2.4E-04

[a] The following analytes had values at or below the lower detection level for the majorities of infected and non-infected animals: Fibroblast Growth Factor 9, Granulocyte Macrophage Colony Stimulating Factor, Interleukin-21 alpha, Interleukin-2, Interleukin-3, Interleukin-4, Interleukin-7, Interleukin-11, Interleukin-12 subunit p70, Interleukin-18, Oncostatin M, and Tumor Necrosis Factor alpha. Fibrinogen was not reported because the sample was plasma.

[b] Bonferroni correction *p* value for mutliple testing for 45 tests and alpha of 0.05: 1.1E-03.
