# Supplementary material for: Elevated Carbon Monoxide in the Exhaled Breath of Mice during a Systemic Bacterial Infection
Source: PLoS One. 2013 Jul 31;8(7):e69802. doi: 10.1371/journal.pone.0069802 (PMC3729689; doi:10.1371/journal.pone.0069802)
Supplement: Table S2 — Carbon monoxide and volatile organic compounds in exhaled breath of mice, in parts per trillion by volume (pptv), except as noted. (PDF) [file pone.0069802.s004.pdf]

Table S2. Carbon monoxide and volatile organic compounds in exhaled breath of mice, in parts per trillion by volume (pptv), except as noted

Compound	CAS registry number	Uninfected - Days -4, -2, and 0 (n = 24 groups of 3)			Infected - Day 5 (n = 8 groups of 3)			*t* test *p* value[b]
		Mean	95% confidence limits[a]		Mean	95% confidence limits		
Carbon monoxide (ppbv)	630-08-0	117	113	126	196	179	229	7.3E-14
Methane (ppmv)	74-82-8	1.83	1.83	1.84	1.83	1.83	1.84	3.0E-01
Ethane	74-84-0	617	568	716	601	565	673	7.2E-01
Propane	74-98-6	418	405	445	408	379	465	4.7E-01
n-Butane	106-97-8	62	47	92	95	86	111	2.1E-02
n-Pentane	109-66-0	62	57	73	65	55	87	5.7E-01
n-Hexane	110-54-3	24	19	36	17	12	25	1.5E-01
i-Pentane	78-78-4	31	11	70	24	17	37	7.0E-01
Ethene	74-85-1	84	70	114	88	62	139	8.2E-01
1-Butene/i-Butene	106-98-9/115-11-7	166	153	193	169	143	220	8.7E-01
Isoprene	78-79-5	13	8	24	6	5	9	1.7E-01
alpha-Pinene	80-56-8	3.7	2.7	5.8	5.7	3.6	9.9	8.3E-02
Benzene	71-43-2	25	23	30	24	21	30	6.5E-01
Toluene	108-88-3	23	19	31	22	18	31	8.5E-01
Ethylbenzene	100-41-4	9	5	17	7	6	9	5.8E-01
m/p-Xylene	108-38-3/106-42-3	26	17	46	23	20	31	7.4E-01
o-Xylene	95-47-6	18	11	31	14	10	23	6.1E-01
n-Propylbenzene	103-65-1	3.0	2.3	4.5	2.8	2.2	4.0	7.1E-01
i-Propylbenzene	98-82-8	2.5	1.1	5.3	1.6	1.4	2.0	4.8E-01
1,2,4-Trimethylbenzene	95-63-6	21.1	11.8	39.9	15.6	13.0	20.9	5.2E-01
1,3,5-Trimethylbenzene	108-67-8	6.7	5.4	9.1	6.7	4.9	10.3	9.7E-01
3-Ethyltoluene	620-14-4	7.3	5.9	10.2	6.4	4.9	9.3	4.9E-01
2-Ethyltoluene	611-14-3	3.6	2.7	5.2	2.8	2.5	3.4	3.1E-01
Methanol	67-56-1	10579	8860	14016	5667	4777	7448	3.6E-03
Ethanol	64-17-5	4341	3365	6293	1735	1023	3158	6.7E-03
Isopropanol	67-63-0	528356	396221	792625	295771	203298	480718	6.4E-02
1-Butanol	71-36-3	5475	3391	9645	2742	1707	4811	1.6E-01
Acetaldehyde	75-07-0	1399	1259	1681	1106	1026	1266	2.9E-02
Butanal	123-72-8	896	491	1706	433	312	673	2.1E-01
2-Methylbutanal	96-17-3	55	49	67	49	44	57	2.2E-01
Pentanal	110-62-3	160	130	220	132	107	181	3.2E-01
Hexanal	66-25-1	604	371	1070	335	167	670	2.2E-01
Acetone	67-64-1	31189	23199	47168	24896	20846	32997	3.9E-01
Butanone	78-93-3	312	250	435	282	251	344	6.0E-01
2-Pentanone	107-87-9	221	135	394	164	129	235	4.7E-01
3-Pentanone	96-22-0	53	46	67	42	38	52	1.1E-01
Methyl isobutyl ketone	108-10-1	82	66	113	56	40	88	9.4E-02
Methyl nitrate	598-58-3	15	14	15	14	14	15	3.6E-01
Ethyl nitrate	625-58-1	10	10	11	10	10	10	1.0E-01
n-Propyl nitrate	627-13-4	2.4	2.4	2.6	2.2	2.2	2.3	1.8E-02
i-Propyl nitrate	1712-64-7	12	11	12	11	11	12	4.0E-02
2-Butyl Nitrate	543-29-3	7.0	6.7	7.6	6.6	6.4	7.0	2.0E-01
2-Pentyl nitrate	21981-48-6	1.6	1.4	2.0	1.3	1.2	1.5	1.6E-01
3-Pentyl nitrate	82944-59-0	1.0	0.9	1.3	0.9	0.8	1.1	1.7E-01
3-Methyl-2-butyl nitrate	Not assigned	3.4	2.3	5.8	2.5	1.5	4.4	3.8E-01
Carbonyl sulfide	463-58-1	458	418	538	374	356	409	2.6E-02
Carbon disulfide	75-15-0	0.27	0.23	0.35	0.28	0.26	0.33	6.9E-01
Dimethyl sulfide	75-18-3	13	9	21	10	4	21	3.8E-01
Dimethyl disulfide	624-92-0	46	30	78	67	0	278	5.2E-01
Dimethyl trisulfide	3658-80-8	28126	15876	52626	31575	14793	65138	7.7E-01
Methyl chloride	74-87-3	869	842	925	867	816	968	9.3E-01
Methyl bromide	74-83-9	8.6	8.3	9.2	7.9	7.6	8.5	1.0E-02
Methyl iodide	74-88-4	0.81	0.69	1.06	0.79	0.67	1.04	8.6E-01
Dichloromethane	75-09-2	30	24	43	26	26	27	4.9E-01
Dibromometane	74-95-3	0.66	0.61	0.76	0.76	0.72	0.85	3.3E-02
Dibromochloromethane	124-48-1	0.68	0.39	1.27	0.70	0.49	1.12	9.6E-01
Bromodichloromethane	75-27-4	0.56	0.29	1.09	0.49	0.30	0.86	7.8E-01
Tribromomethane	75-25-2	1.03	0.93	1.23	0.99	0.93	1.12	7.0E-01
Trichloromethane	67-66-3	70	55	99	49	39	68	1.3E-01
Trichloroethene	79-01-6	1.05	0.97	1.22	1.15	1.01	1.43	2.6E-01
Tetrachloroethene	127-18-4	4.2	3.7	5.2	3.4	3.1	4.1	1.1E-01
Tetrachloromethane	56-23-5	90	88	95	89	88	91	6.3E-01
1,1,1-Trichloroethane	71-55-6	9	9	10	9	8	10	8.5E-01
Ethyl chloride (area)	75-00-3	1019	959	1141	829	670	1149	1.1E-02
Dichlorodifluorometane	75-71-8	541	536	552	549	545	557	1.2E-01
Trichlorofluoromethane	75-69-4	262	258	271	258	257	261	2.9E-01
Trichlorotrifluoroethane	76-13-1	80	79	84	79	79	80	3.9E-01
1,2-Dichlorotetrafluoroethane	76-14-2	16.6	16.1	17.6	17.3	16.7	18.5	1.4E-01
Bromochlorodifluoromethane	353-59-3	4.7	4.6	5.0	4.7	4.5	5.0	7.9E-01
1,1-Dichloro-1-fluoroethane	1717-00-6	22	22	24	21	21	22	2.3E-01
1-Chloro-1,1-difluoroethane	75-68-3	22	21	23	21	20	21	6.3E-02
Chlorodifluoromethane	75-45-6	228	223	239	223	220	230	3.0E-01
1,1,1,2-Tetrafluoroethane	811-97-2	52	51	55	51	48	55	1.5E-01

[a] Asymmetric antilogs of 95% confidence limits determined on log-transformed values.
[b] Bonferroni correction *p* value for mutliple testing for 73 tests and alpha of 0.05: 7E-04.
